# Anesthesia, analgesia, and immunonutrition in perioperative immune homeostasis: a focused mini review

**DOI:** 10.3389/fnut.2026.1938301

**Published:** 2026-09-18

**Authors:** Ran Lan, Jiali Yao, Lei Zhang

**Affiliations:** 1 Department of Anesthesiology, Jinhua Hospital Affiliated to Zhejiang University, Jinhua, Zhejiang, China; 2 Department of Critical Care Medicine, Jinhua Hospital Affiliated to Zhejiang University, Jinhua, Zhejiang, China

**Keywords:** anesthesia, gut barrier, immunonutrition, inflammation, opioid-sparing analgesia, perioperative immunity, regional anesthesia, surgical oncology

## Abstract

Surgical injury compresses tissue damage, neuroendocrine stress, pain, fasting, microbial exposure, and repair into a brief interval of intense immune activity. Although anesthetic, analgesic, and nutritional care are usually examined separately, each can affect cytokine release, immune-cell trafficking, oxidative stress, macrophage activity, gut barrier integrity, substrate availability, and inflammatory resolution. This mini review examines the mechanistic and clinical evidence linking anesthetic technique, regional and opioid-sparing analgesia, anti-inflammatory adjuncts, and immunonutrition with perioperative immune homeostasis, focusing on major gastrointestinal and cancer surgery. Current evidence indicates that anesthetic and analgesic strategies can alter inflammatory or immune-cell phenotypes, and these changes are most informative when interpreted as surrogate or mechanistic endpoints linked to patient-important outcomes. Immunonutrition has the clearest clinical evidence in gastrointestinal cancer surgery, particularly among nutritionally vulnerable or high-risk patients, although effects vary with formula composition, timing, route, adherence, procedure, and comparator. Direct trials of integrated anesthesia-analgesia-immunonutrition pathways are now needed to connect these mechanistic pathways with recovery outcomes. Risk-stratified studies can use this framework to examine both component-specific effects and interactions across the wider pathway.

## 1 Introduction

Surgery combines a controlled tissue injury with an intense repair response. Tissue damage, ischemia–reperfusion, pain, blood loss, hypothermia, and microbial exposure activate innate immune signaling and neuroendocrine stress pathways (1). When appropriately regulated, this response supports hemostasis, antimicrobial defense, wound repair, and resolution. When excessive or prolonged, it may instead promote immune dysfunction, infection, organ injury, persistent catabolism, and delayed recovery (2). In major surgery, the pace of inflammatory resolution has tangible consequences for feeding, mobilization, postoperative infection, and timely return to planned oncologic treatment.

Several routine perioperative decisions intersect with this biology. Anesthetic and analgesic choices can influence sympathetic activation, hypothalamic–pituitary–adrenal signaling, cytokine release, natural killer (NK) cell activity, lymphocyte subsets, macrophage polarization, oxidative stress, and pain-driven inflammation (3). Immunonutrition acts through a different but overlapping set of processes, including immune-cell metabolism, nitrogen balance, membrane lipid composition, eicosanoid biology, specialized pro-resolving mediator production, and mucosal defense (4, 5). This review asks how anesthesia, analgesia, and immunonutrition might jointly modify perioperative immune trajectories in patients with different levels of surgical and nutritional risk. Its aim is to distinguish mechanistic signals from clinical outcomes and identify study designs that could test integrated immunometabolic care in major gastrointestinal or cancer surgery (Table 1).

**Table 1:** Evidence hierarchy for perioperative immunometabolic interventions.

Evidence domain	Population and study design	Comparator	Intervention details to report	Outcome level	Certainty	Key limitations
Volatile vs. intravenous anesthesia (12–24)	Thoracic, breast, colorectal, oral, and esophageal surgery; mainly RCTs and small mechanistic clinical studies	Volatile-based general anesthesia vs. propofol/TIVA or mixed regimens	Agent, dose, depth, ventilation, temperature, blood loss, transfusion, steroids, analgesia	Mostly cytokines, oxidative stress, NK activity, lymphocyte subsets, immune-cell profiles; limited patient-important outcomes	Low to moderate for biomarkers; low for clinical outcomes	Procedure heterogeneity; small single-center studies; co-analgesia and perioperative care often incompletely reported
Neuraxial and regional analgesia (25–29, 51, 52, 56, 60)	Major abdominal, colon cancer, lung cancer, breast cancer, spine, and orthopedic surgery; RCTs, observational studies, reviews	General anesthesia or systemic analgesia without the regional technique	Epidural/spinal/paravertebral/fascial plane block or wound infiltration; success, duration, local anesthetic dose, rescue opioids	Pain, opioid exposure, stress markers, lymphocyte function, gut recovery; recurrence/survival evidence remains inconsistent	Moderate for analgesic effect; low to moderate for immune biomarkers; low for oncologic outcomes	Block failure, hypotension, anticoagulation constraints, institutional expertise, and ERAS adherence confound interpretation
Opioid-sparing or opioid-free anesthesia and adjuncts (30–32, 50, 53, 54)	Gastric cancer, block-based perioperative analgesia, and mechanistic adjunct literature; early RCTs and narrative/scoping reviews	Conventional opioid-based anesthesia or usual multimodal analgesia	Morphine-equivalent exposure, rescue analgesia, ketamine/esketamine, lidocaine dose and infusion duration, regional block use	Inflammatory factors, macrophage polarization, pain scores, opioid dose, ileus, feeding tolerance, adverse events	Low to moderate for short-term biomarkers; low for durable clinical outcomes	Opioid-free and opioid-sparing strategies are often conflated; effect modification by neoadjuvant therapy is untested
Enteral/oral immunonutrition formulas (33–38, 43–49, 57, 58, 62, 63)	Gastrointestinal cancer, gastric cancer, esophagectomy, pancreaticoduodenectomy, radical cystectomy, and high-risk surgery; RCTs, systematic reviews, meta-analyses	Standard nutrition, standard enteral formula, usual care, or no immune-enriched formula	Arginine, omega-3 fatty acids, glutamine, nucleotides, antioxidants; route, timing, duration, dose, adherence, interruptions	Infections, complications, barrier markers, immune indices, length of stay, tolerance/intake, cost-effectiveness	Moderate in selected GI/high-risk populations; low for universal use	Nutritional risk, cancer stage, neoadjuvant treatment, procedure, comparator, adherence, and ERAS care vary
Omega-3/fish-oil strategies (39, 40, 62)	Major gastrointestinal surgery and parenteral/enteral nutrition literature; systematic reviews and mechanistic nutrition studies	Standard lipid or non-omega-3 nutrition support	EPA/DHA dose, oral/enteral/parenteral route, start date, duration, background formula composition	Eicosanoid balance, specialized pro-resolving mediator biology, cytokine tone, complications in selected studies	Low to moderate	Dose, formulation, route, and perioperative timing vary; clinical endpoints are not uniformly defined
Integrated anesthesia-analgesia-immunonutrition pathway (64)	No dedicated adequately controlled trial testing the combined immune-metabolic pathway; ERAS literature provides implementation context	Standard ERAS or usual perioperative care with isolated anesthetic or nutrition components	Prespecified anesthetic regimen, regional technique, opioid exposure, nutrition formula, adherence, feeding route, and rescue pathways	Clinical recovery plus prespecified immune improvement, immune null effect, or immune change without clinical benefit	Hypothesis-generating	Not clinically validated; coordination, contamination, adherence, confounding, and interaction sample size complicate trials

NK, natural killer; RCT, randomized controlled trial. Evidence certainty reflects the consistency, design quality, and clinical relevance of the cited perioperative literature, not a formal GRADE assessment.

## 2 Search strategy and evidence selection

This mini review used a narrative evidence-selection strategy. PubMed was searched through July 2026 using terms related to anesthesia, analgesia, regional anesthesia, opioid-sparing or opioid-free anesthesia, lidocaine, ketamine, immunonutrition, arginine, omega-3 fatty acids, glutamine, nucleotides, antioxidants, perioperative immunity, inflammation, gut barrier, postoperative complications, enhanced recovery, and cancer surgery. Priority was given to randomized trials, systematic reviews, meta-analyses, perioperative nutrition guidance, and clinically relevant gastrointestinal or oncologic surgery studies. Mechanistic studies were included when they clarified immune-cell phenotypes, cytokine responses, macrophage polarization, oxidative stress, inflammatory resolution, or barrier biology. Evidence selection was narrative rather than systematic; accordingly, this review provides a qualitative synthesis rather than a quantitative estimate of clinical effectiveness. Biomarkers are discussed as surrogate or mechanistic endpoints unless supported by direct outcome evidence.

## 3 Perioperative immune trajectories across surgical stress, cancer risk, and host reserve

Perioperative immunity evolves over time. Cytokines, complement, coagulation, leukocyte trafficking, and acute-phase responses are mobilized soon after injury to contain damage and initiate repair. Poor resolution can be followed by lymphocyte dysfunction, impaired antigen presentation, barrier failure, and greater susceptibility to infection. Because these events unfold on different timescales, an immediate cytokine rise, an early postoperative lymphocyte fall, and a later barrier abnormality may describe successive phases rather than conflicting findings. Clinical studies have documented rapid changes in circulating lymphocyte subpopulations during opioid-based general anesthesia (6) and reductions in immune-cell populations during craniotomy (7), showing that measurable immune availability can shift within the surgical-anesthetic interval. Repeated sampling from induction through the first postoperative days is therefore more informative than a single biomarker value, particularly when the clinical question concerns resolution rather than the initial inflammatory peak.

In cancer surgery, this transient immune disturbance coincides with wound-healing signals, possible residual microscopic disease or circulating tumor cells, and interruptions in systemic therapy. Reviews of perioperative anesthesia have consequently examined immune surveillance, inflammatory mediators, and angiogenic signaling as potential links to tumor progression (8). Systematic reviews comparing inhalational and intravenous anesthesia provide clinical context but do not establish a consistent long-term advantage for either technique (9). Recent cancer-focused reviews likewise place recurrence and metastasis within a wider setting that includes tumor stage and biology, surgical quality, systemic therapy, postoperative complications, and host reserve (10). Perioperative immune disturbance is one potentially modifiable part of that setting, not an independent determinant of recurrence or survival. We propose treating an immune measure as a candidate surrogate only when its assay and sampling are reproducible, it tracks a prespecified clinical event longitudinally and adds information beyond baseline risk, and intervention-related differences in the measure correspond to differences in that event across studies. Patient-level association alone is insufficient; without trial-level validation, the measure remains a mechanistic endpoint.

Host reserve also changes the response to a comparable surgical insult. Malnutrition, sarcopenia, insulin resistance, cachexia, liver disease, dysbiosis, advanced age, and poor oral intake can limit the substrates required for immune-cell proliferation, mediator synthesis, epithelial repair, and barrier defense (11). Differences in baseline reserve, together with inconsistent sampling schedules, may account for some of the variation in perioperative biomarkers across studies. A phase-based interpretation therefore matters: intraoperative markers alone provide an incomplete view of anesthetic effects, while late complications after immunonutrition are difficult to interpret without information on anesthesia, analgesia, early feeding, and the preceding immune trajectory.

## 4 Anesthetic technique as a context-dependent modulator of inflammatory and cellular immune phenotypes

Comparative anesthesia trials detect immune effects, but the direction and magnitude are not uniform across operations. Volatile and intravenous techniques have produced different alveolar and systemic inflammatory responses in thoracic surgery (12), and other thoracic studies have reported differences in oxidative stress during propofol and sevoflurane anesthesia (13). During one-lung ventilation, pulmonary inflammation may interact with the anesthetic exposure itself (14). Technique-related changes in postoperative inflammatory markers have also been reported outside cancer surgery, including after microdiscectomy (15). Across these settings, one-lung ventilation, mucosal contamination, pneumoperitoneum, tumor manipulation, and blood loss may equal or exceed the signal attributed to a particular anesthetic. Interpretation therefore depends on complete reporting of the anesthetic regimen, adjunct drugs, ventilation, temperature, fluids, transfusion, and postoperative analgesia.

Cancer-surgery trials increasingly include more specific immune phenotyping. In breast cancer surgery, anesthesia-analgesia combinations have been studied in relation to perioperative immune function (16), including serum NK-cell antitumor activity as a functional endpoint (17). Other studies have measured cytokines and matrix metalloproteinases (18), compared immune responses across anesthetic techniques (19), or examined neutrophil-lymphocyte ratio, platelet-lymphocyte ratio, and return to intended oncologic therapy (20). In colorectal cancer surgery, immune-cell expression profiling after propofol or sevoflurane anesthesia extended the analysis beyond isolated cytokine measurements (21). Multidimensional phenotyping can reveal whether several immune compartments move together; its clinical value depends on linkage to patient outcomes. Longitudinal profiles are especially valuable when they connect an early cellular change with later infection, gut recovery, treatment delay, or another prespecified outcome in the same patient.

Recent oral cancer studies have compared general anesthetic techniques during flap reconstruction (22) and examined transforming growth factor-beta as a marker related to antitumor immunity (23). Esophageal cancer surgery has provided another setting in which cellular immune function and inflammatory responses were measured under specific anesthetic regimens (24). Across these studies, the observed phenotype remains inseparable from cancer type, procedure, sampling schedule, co-analgesia, ventilation, temperature, blood loss, steroid use, and the broader recovery pathway. Technique-related biomarkers are most informative when these factors are reported and when sampling is linked prospectively to clinical recovery.

## 5 Analgesic strategy, regional techniques, and opioid burden as immune-relevant recovery levers

Analgesic management also shapes the perioperative stress response. Pain increases sympathetic and endocrine activation, whereas regional and neuraxial techniques can reduce nociceptive input and opioid exposure. Epidural approaches have been associated with preserved lymphocyte function after major spine surgery (25) and attenuated stress-induced immunosuppression during major abdominal surgery (26). In fast-track colon cancer surgery, combined general and epidural anesthesia was associated with less immunosuppression and earlier intestinal functional recovery (27). Studies in lung cancer (28) and orthopedic surgery (29) suggest that immune effects are not confined to colorectal procedures. Their relevance, however, depends on the technique and operation: thoracic epidural analgesia, spinal anesthesia, paravertebral or fascial plane blocks, truncal blocks, wound infiltration, intravenous lidocaine, ketamine or esketamine, and opioid-free anesthesia do not share the same efficacy, risks, or evidence base. Hypotension, block failure, local anesthetic toxicity, anticoagulation constraints, and institutional expertise all influence net clinical value. Immune measures are therefore best considered alongside analgesic quality, safety, opioid exposure, ileus, feeding tolerance, and implementation fidelity.

Early studies have begun to examine opioid-sparing pathways more directly. In gastric cancer patients treated with neoadjuvant PD-1 inhibition, opioid-free anesthesia was associated with less perioperative immunosuppression and differences in macrophage polarization (30). This study did not compare the intervention effect with that in treatment-naive patients; current evidence therefore cannot determine whether neoadjuvant immunotherapy, chemotherapy, or radiotherapy modifies efficacy. Future trials should stratify by neoadjuvant modality, timing, and response and prespecify treatment-by-intervention interactions. A randomized study of a quadratus lumborum block-based opioid-free regimen found that adding esketamine reduced selected inflammatory factors (31), while the effects of intravenous lidocaine vary with procedure, dose, infusion duration, and endpoint (32). These findings support a mechanistic rationale, but opioid-free anesthesia remains distinct from rational opioid-sparing analgesia. Complete avoidance may still lead to rescue analgesia, whereas a multimodal pathway can reduce cumulative exposure without making avoidance the overriding goal. Reporting morphine-equivalent exposure, pain, block success, rescue treatment, ileus, feeding tolerance, adverse effects, and protocol deviations would make immune outcomes easier to interpret.

## 6 Immunonutrition as targeted immune-metabolic conditioning for nutritionally vulnerable surgical patients

Immunonutrition is an umbrella term for formulas enriched with arginine, omega-3 fatty acids, glutamine, nucleotides, antioxidants, or combinations of these substrates. Their biological roles differ: arginine-containing formulas may support T-cell activity and nitric oxide biology; omega-3 products may alter eicosanoid and specialized pro-resolving mediator pathways; glutamine contributes to enterocyte metabolism and barrier repair; nucleotides may support proliferating cells; and antioxidants are intended to limit oxidative stress. Meta-analyses in gastrointestinal cancer surgery suggest improvement in selected postoperative outcomes (33), while evidence maps in gastric cancer (34), esophagectomy-focused analyses (35), and randomized-trial syntheses across gastrointestinal procedures (36) show substantial heterogeneity. The clinical signal depends on what the formula contains, who receives it, and when and how it is delivered.

More recent studies add detail to this heterogeneous evidence base. Immunomodulatory enteral nutrition has been associated with clinical, biochemical, and nutritional changes after gastrointestinal cancer surgery (37), and a randomized gastrectomy study linked perioperative enteral immunonutrition with immune function and intestinal mucosal barrier measures (38). Omega-3 fatty acids connect nutrition with lipid-mediator biology; reviews of preoperative n-3 fatty acids (39) and fish-oil-based nutrition (40) describe potential inflammatory effects that vary by dose, route, and timing. Broader reviews continue to identify formula composition, patient selection, and outcome choice as major sources of variation (41). Evidence outside gastrointestinal surgery remains less developed, although ovarian cancer studies suggest another candidate population (42). Preoperative oral supplementation in a malnourished patient is not equivalent to postoperative enteral feeding, parenteral fish-oil lipid emulsions, or late supplementation in a septic patient. Upper gastrointestinal, colorectal, pancreatic, urologic, and gynecologic procedures also differ in nutritional risk, enteral tolerance, contamination risk, complication profile, and likelihood of delayed oncologic therapy.

Clinical use is most plausible when nutritional support is matched to postoperative risk (43). Immunonutrition may be particularly relevant when gut function is threatened (44), and surgical nutrition reviews favor considering the active substrates rather than treating all enriched formulas as equivalent (45). Patients undergoing radical cystectomy (46), other high-risk operations (47), or pancreaticoduodenectomy (48) may combine high immune demand with limited substrate reserve, and cost-effectiveness analyses favor concentrating resources in patients with sufficient baseline risk (49). Heterogeneity is driven by nutritional status and sarcopenia, cancer site and stage, neoadjuvant treatment, procedure and surgical approach, formula and comparator composition, timing, route, dose, adherence, background ERAS care, and postoperative complications. Future trials should stratify randomization by nutritional risk and procedure, standardize the comparator and background care, record actual intake and interruptions, and prespecify clinically plausible subgroup interactions. Delivery failure from nausea, ileus, interruption, poor adherence, or an unsuitable route should not be interpreted as biological failure.

## 7 Timing, gut barrier function, and integrated pathway design across the perioperative window

Timing offers one explanation for apparently inconsistent findings. Before surgery, nutritional depletion and systemic inflammation define immune reserve. During surgery, anesthetic exposure, nociceptive signaling, hemodynamic instability, hypothermia, transfusion, vasopressor use, ventilation, fluids, glycemic control, steroids, antibiotics, and tissue handling shape the early inflammatory peak. After surgery, pain, ileus, opioids, fasting, infection, delayed mobilization, surgical approach, and adherence to enhanced recovery determine whether inflammation resolves or persists. Opioid-sparing anesthesia is most likely to matter when it improves analgesia and recovery rather than functioning only as a pharmacologic label (50). Regional techniques must similarly be understood within the stress response and the wider recovery pathway (51, 52). Lidocaine has plausible anti-inflammatory effects (53), and experimental work suggests that some anesthetic exposures may also influence resolution biology (54). Nutrition is equally time-sensitive: a formula given too late, too briefly, or to a patient unable to absorb it is unlikely to alter recovery. An integrated pathway therefore needs explicit timing for preoperative nutrition, intraoperative stress control, postoperative analgesic stewardship, and early feeding. Figure 1 illustrates this conceptual, hypothesis-generating framework and includes the possibility that a measurable immune change occurs without detectable clinical benefit.

**Figure 1:**
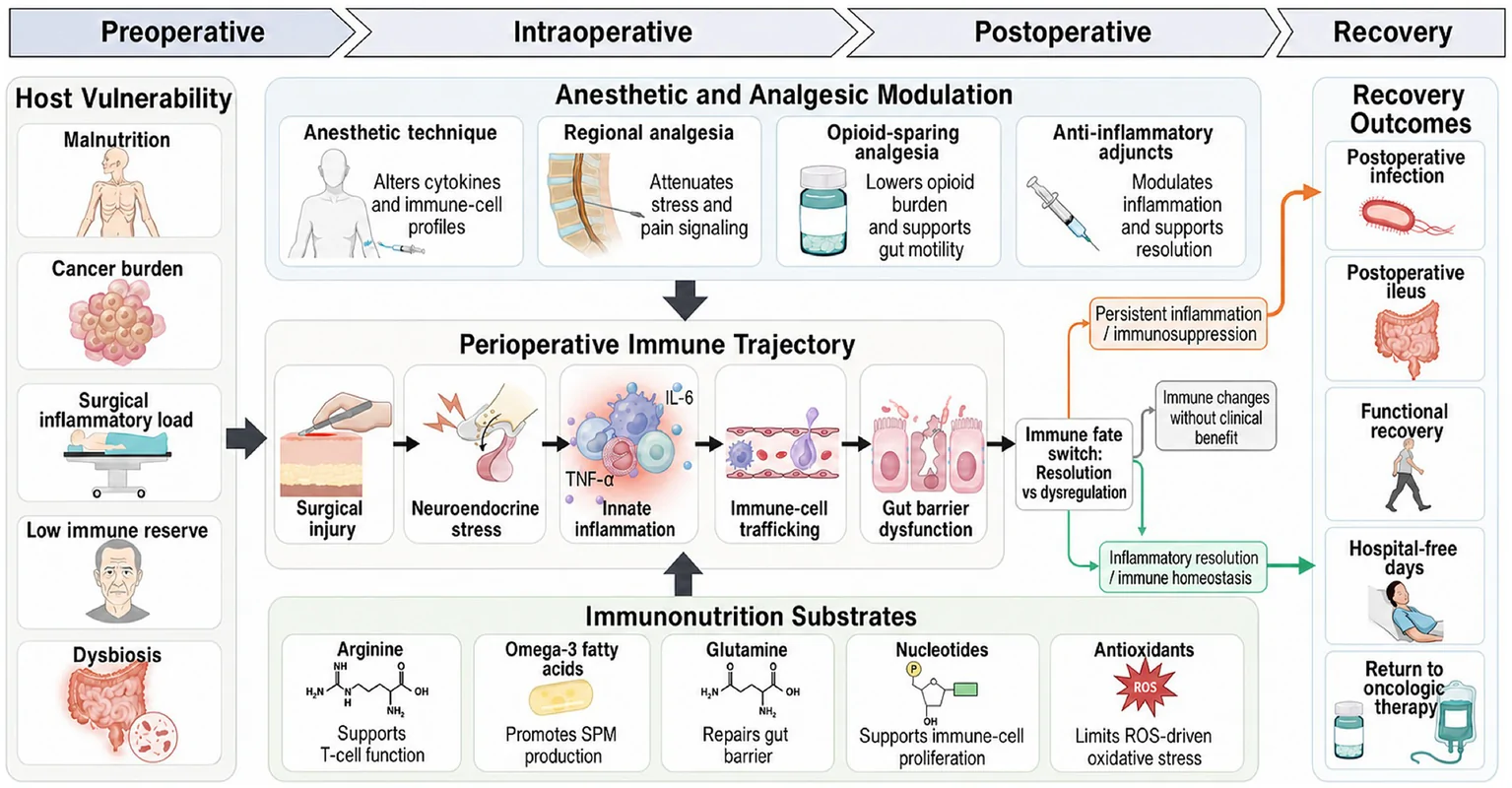
Conceptual immunometabolic framework linking anesthesia, analgesia, immunonutrition, perioperative immune trajectories, and postoperative recovery. Host vulnerability, including malnutrition, cancer burden, surgical inflammatory load, low immune reserve, and dysbiosis, may shape the host response to surgical injury. Surgical injury may trigger neuroendocrine stress, innate inflammation, immune-cell trafficking, and gut barrier dysfunction, thereby shifting the perioperative immune trajectory toward persistent inflammation/immunosuppression, inflammatory resolution/immune homeostasis, or a measurable immune change without detectable clinical benefit. Anesthetic and analgesic strategies may modulate this trajectory by altering cytokines and immune-cell profiles, attenuating stress and pain signaling, lowering opioid burden, supporting gut motility, and promoting inflammatory resolution. Immunonutrition substrates, including arginine, omega-3 fatty acids, glutamine, nucleotides, and antioxidants, may support T-cell function, specialized pro-resolving mediator production, gut barrier repair, immune-cell proliferation, and control of ROS-driven oxidative stress. These immunometabolic processes are proposed to be clinically relevant to postoperative infection, postoperative ileus, functional recovery, hospital-free days, and return to oncologic therapy, and should be tested prospectively in integrated perioperative pathways. The gray branch denotes a biomarker or immune change without a corresponding improvement in patient-important outcomes. Arrows indicate hypothesized directional influence and do not imply independent clinical superiority of any single anesthetic, analgesic, or nutritional intervention. IL-6, interleukin-6; TNF-α, tumor necrosis factor-α; ROS, reactive oxygen species; SPM, specialized pro-resolving mediator.

The gut barrier provides the clearest point of contact between analgesia and nutrition. Opioids, ileus, fasting, antibiotics, dysbiosis, and major abdominal surgery can impair enteral tolerance and mucosal defense. Immune-enriched nutrition may support epithelial repair, while effective regional or opioid-sparing analgesia may facilitate motility and feeding. Reviews of anesthetic technique and cancer recurrence remain cautious (55), and comparisons of regional with general anesthesia support measuring immune pathways alongside long-term outcomes (56). In upper gastrointestinal cancer surgery, enteral immunonutrition has been compared with standard nutrition (57), while earlier reviews emphasized postoperative complications rather than biomarkers alone (58). Circulating markers of epithelial injury or microbial translocation can support temporal profiling, but they do not establish mucosal integrity or microbiome function. Trials should prioritize clinical gut recovery, including ileus, tolerance of oral or enteral intake, and time to first stool; add permeability testing and direct mucosal assessment where feasible; and use serial stool sampling for microbiome composition and functional analysis. Agreement across clinical, circulating, mucosal, and microbiome measures is more informative than any single marker.

Risk stratification gives an integrated pathway a clinically useful starting point. Well-nourished patients at low surgical risk may require no more than standard enhanced recovery care, effective analgesia, early mobilization, and early oral intake. Patients undergoing esophagectomy, gastrectomy, pancreaticoduodenectomy, colorectal resection, or major oncologic surgery after neoadjuvant therapy often face a different combination of nutritional depletion, inflammatory burden, and delayed gut recovery. In these patients, nutritional screening, short-course immunonutrition when feasible, opioid-sparing multimodal analgesia, regional or neuraxial techniques when safe, and monitoring of infection, ileus, hospital-free days, and return to intended oncologic therapy can be studied as a coordinated pathway. Oesophagogastric and gastrointestinal cancer nutrition literature supports focusing this work on complication-prone populations (57, 58). Prespecified rescue criteria are also needed for failed oral intake, block failure, escalating opioid requirements, infection, and delayed mobilization.

## 8 Discussion

Anesthesia, analgesia, and immunonutrition occupy different parts of the same perioperative immune environment, but no direct trial has shown that aligning them improves recovery through an immune mechanism. Long-term oncologic reviews argue against claiming that one anesthetic technique reliably prevents recurrence (59), and randomized evidence in breast cancer surgery is similarly cautious (60). Tumor heterogeneity, systemic therapy, surgical quality, complications, and incomplete perioperative reporting make causal interpretation difficult (61). Joint trials add further obstacles: several clinical teams must coordinate timing and adherence, component regimens vary, blinding is limited, usual care can contaminate contrasts, and interaction effects require larger samples. Component-specific practices such as nutritional screening, indicated regional or multimodal analgesia, opioid stewardship, and early feeding should therefore follow their own evidence and clinical indications; current evidence does not justify prescribing the combined pathway for an unproven immune benefit. Pragmatic factorial trials could test component effects and interactions while retaining standard ERAS care.

Generalizability of immunonutrition findings depends largely on who was treated and how the formula was delivered. A gastrointestinal-surgery signal is more clinically useful when nutritional vulnerability, start time, substrate composition, dose, route, adherence, and the intended clinical outcome are all defined. Recent reviews of immune- and metabolic-modulating nutrition support this pathway-based approach (62), and expert recommendations emphasize structured perioperative nutritional assessment and delivery (63). Nutritional screening should occur during preoperative planning, before ileus, infection, or delayed adjuvant therapy exposes an unrecognized deficit. Reports also need the same detail for formula composition, duration, compliance, and interruption that anesthesia studies provide for drugs and regional techniques.

A pathway-level trial would be more informative than another isolated biomarker comparison. It would specify the anesthetic regimen, regional technique, opioid exposure, steroid and antibiotic use, nutrition formula, adherence, feeding route, and sampling windows. Lymphocyte subsets, NK-cell function, cytokine signatures, macrophage polarization, gut barrier markers, lipid mediators, and selected microbiome measures could help explain biological response only when linked prospectively to clinical outcomes. Outcomes should be setting-specific and time-defined: 30-day complications and infectious morbidity, early ileus and enteral tolerance, length of stay and 30-day hospital-free days, readmission, functional recovery, nutritional intake and adherence, delay in return to intended oncologic therapy, and recurrence or survival only when adequately powered and controlled. Enhanced recovery evidence shows that bundled care can improve short-term outcomes (64); an immunometabolic pathway must additionally show whether biomarker differences accompany clinically meaningful recovery. Flow-based metabolic profiling, metabolic flux assays, stable-isotope tracing, transcriptomics, and imaging can improve mechanistic resolution (65), but technical depth does not itself confer surrogate validity. A shared minimum dataset would help distinguish biological null effects from poor delivery or adherence.

Perioperative immune homeostasis crosses the usual boundaries between anesthesia, surgery, nutrition, and oncology. Convergence is most plausible when inflammatory burden is high, nutritional reserve is low, opioid requirements are substantial, or gut recovery is delayed. A risk-enriched pathway trial could determine whether coordinated care improves postoperative resilience and whether immune resolution contributes to that effect.
